# RNOP-09: Pegylated liposomal doxorubicine and prolonged temozolomide in addition to radiotherapy in newly diagnosed glioblastoma - a phase II study

**DOI:** 10.1186/1471-2407-9-308

**Published:** 2009-09-02

**Authors:** Christoph P Beier, Christina Schmid, Thierry Gorlia, Christine Kleinletzenberger, Dagmar Beier, Oliver Grauer, Andreas Steinbrecher, Birgit Hirschmann, Alexander Brawanski, Christopher Dietmaier, Tanja Jauch-Worley, Oliver Kölbl, Torsten Pietsch, Martin Proescholdt, Petra Rümmele, Armin Muigg, Günther Stockhammer, Monika Hegi, Ulrich Bogdahn, Peter Hau

**Affiliations:** 1 Department of Neurology, University of Regensburg, Universitätsstrasse 84, 93053 Regensburg, Germany; 2 EORTC Data Center, Avenue Mounierlaan 83/11, 1200, Brussels, Belgium; 3 Department of Neurosurgery, University of Regensburg, Franz-Josef-Strauss Allee 11, 93053 Regensburg, Germany; 4 University of Applied Sciences Amberg Weiden, Hetzenrichter Weg 15, 92224 Weiden, Germany; 5 Department of Radiooncology, University of Regensburg, Franz-Josef-Strauss Allee 11, 93053 Regensburg, Germany; 6 Department of Neuropathology, University of Bonn, Sigmund-Freud-Strasse 3, 53015 Bonn, Germany; 7 Department of Pathology, Franz-Josef-Strauss Allee 11, 93053 Regensburg, Germany; 8 Department of Neurology, University of Innsbruck, Anichstrasse 35, 6020, Innsbruck, Austria; 9 Laboratory of Brain Tumor Biology and Genetics, Centre Universitaire Romands de Neurochirurgie and University of Lausanne, Rue du Bugnon 46, 1011 Lausanne, Switzerland

## Abstract

**Background:**

Although Temozolomide is effective against glioblastoma, the prognosis remains dismal and new regimens with synergistic activity are sought for.

**Methods:**

In this phase-I/II trial, pegylated liposomal doxorubicin (Caelyx™, PEG-Dox) and prolonged administration of Temozolomide in addition to radiotherapy was investigated in 63 patients with newly diagnosed glioblastoma. In phase-I, PEG-Dox was administered in a 3-by-3 dose-escalation regimen. In phase-II, 20 mg/m^2 ^PEG-Dox was given once prior to radiotherapy and on days 1 and 15 of each 28-day cycle starting 4 weeks after radiotherapy. Temozolomide was given in a dose of 75 mg/m^2 ^daily during radiotherapy (60 Gy) and 150-200 mg/m^2 ^on days 1-5 of each 28-day cycle for 12 cycles or until disease progression.

**Results:**

The toxicity of the combination of PEG-Dox, prolonged administration of Temozolomide, and radiotherapy was tolerable. The progression free survival after 12 months (PFS-12) was 30.2%, the median overall survival was 17.6 months in all patients including the ones from Phase-I. None of the endpoints differed significantly from the EORTC26981/NCIC-CE.3 data in a post-hoc statistical comparison.

**Conclusion:**

Together, the investigated combination is tolerable and feasible. Neither the addition of PEG-Dox nor the prolonged administration of Temozolomide resulted in a meaningful improvement of the patient's outcome as compared to the EORTC26981/NCIC-CE.3 data

**Trial registration:**

clinicaltrials.gov NCT00944801.

## Background

Glioblastomas represent 40% of all tumors of the central nervous system (CNS) and are among the most lethal tumors. Therapy comprising debulking surgery and radiotherapy prolongs the median overall survival after initial diagnosis to only 8-12 months [1,2]. Temozolomide (Temodar™, TMZ) combined with radiotherapy was the first substance to significantly improve the overall survival (to 14.6 months) as compared to surgery and radiotherapy alone and increased the proportion of patients surviving more than 2 years to 26%. TMZ showed the best efficacy in patients with a methylated O^6^-methylguanine-DNA methyltransferase (MGMT) promoter by eliminating more sensitive differentiated tumor cells and in part stem cell-like tumor cells [3,4]. Among patients with a methylated MGMT promoter, the median survival after treatment with combined radio-chemotherapy was 21.7 months, as compared to 15.3 months among those who were assigned to radiotherapy only. In the absence of methylation of the MGMT promoter, there was a smaller and statistically insignificant difference in survival between the treatment groups [4].

Doxorubicin is one of the most effective substances *in vitro *against cells derived from glioblastoma [5]. However, it has no significant effect *in vivo *due to poor blood-brain-barrier penetration [6]. In a tumor model, tissue and CSF-concentrations of doxorubicin were substantially increased when sterically stabilized liposomes were used [7] resulting in a comparable clinical response using approximately half of the dose of stabilized liposomes compared to conventional doxorubicin [8]. A pegylated formulation (PEG-liposomal Doxorubicin, Caelyx™, PEG-Dox) even further improved the penetration of the blood-brain barrier [9]. Case series and two phase II-studies in patients with recurrent glioblastoma have shown modestly promising results for PEG-Dox [10-13]. In our cohort, we treated 27 patients with recurrent glioblastoma with 20 mg/m^2 ^PEG-Dox on days 1 and 15 of each 28-day cycle. The overall response rate was 39%. The progression free survival at 6 and 12 months after initiation of therapy was 15% and 7.5%, and median time to tumor progression for responders was 14 weeks, respectively. Median overall survival was 68 weeks after initial diagnosis and 26 weeks after initiation of the relapse regimen [11].

Based on these results, we combined PEG-Dox, TMZ, and radiotherapy in the study presented here, adapting the standard of care EORTC26981/NCIC-CE.3 protocol. Because long-term administration of TMZ for more than 6 cycles (as used in the EORTC26981/NCIC-CE.3 trial) is feasible and well tolerated [14], we decided to administer TMZ for at least 12 cycles or until disease progression. To determine the dose limiting toxicity of PEG-Dox combined with prolonged administration of TMZ, we performed a phase I part ahead of the phase II study. To investigate, by means of a historical control analysis, if the addition of PEG-Dox to TMZ and radiotherapy improves the survival of patients, we chose similar inclusion criteria and identical TMZ and radiotherapeutic regimes as in the EORTC26981/NCIC-CE.3 study.

## Methods

### Patients and Selection Criteria

**Figure 1:**
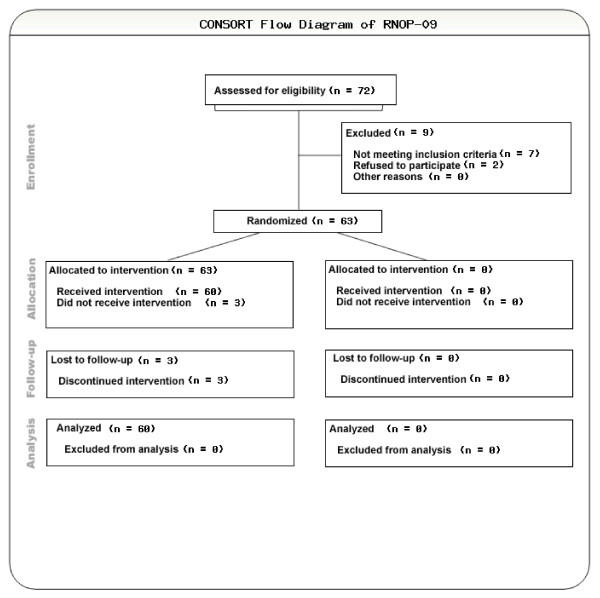
**CONSORT flow diagram**.

From June 2002 until November 2007, 63 patients with newly diagnosed glioblastoma in two neurooncology centers (Department of Neurology, University of Regensburg, Germany and Department of Neurology, University of Innsbruck, Austria) were selected for the study (Figure 1). Eligible patients aged 18 to 70 with centrally confirmed histology (Institute for Neuropathology, University of Bonn, Germany) were included. Inclusion criteria were adapted to the EORTC26981/NCIC-CE.3 study and were, among others, as follows: Karnofsky performance score (KPS) ≥ 70%, stable corticosteroids within 2 weeks before inclusion, leucocytes > 3/ul, thrombocytes > 100/ul, Hb > 10 g/dl. The study was approved by the ethics committees of the participating centers, and was performed in accordance to the applying international regulations. All patients provided written informed consent. The trial was registered at clinicaltrials.gov: NCT00944801.

### Study Design and Treatment

Within 4 weeks after the histological diagnosis of glioblastoma, all patients received standard radiotherapy (total dose 60 Gy; fractions of 2 Gy Monday to Friday) plus concomitant daily TMZ 75 mg/m^2 ^orally daily (including weekends). Radiotherapy was planned with dedicated computed tomography and three-dimensional planning systems and delivered to the gross tumor volume with a 2 to 3 cm margin for the clinical target volume. After a 4-week break, patients received adjuvant TMZ 150 to 200 mg/m^2 ^day 1 to 5 in 28 days until tumor progression or up to at least 12 cycles [2]. In the dose escalation phase of the study, PEG-Dox was raised in steps of 5 mg/m^2 ^in a 3-by-3 design, starting with 5 mg/m^2 ^(group 1) up to 20 mg/m^2 ^(group 4). In the phase II part of the study, the targeted dose of 20 mg/m^2 ^was administered up to a cumulative dose of 550 mg/m^2 ^or until tumor progression.

The following events were defined as dose limiting toxicity (DLT) occurring within the first cycle of adjuvant treatment and graded according to the National Cancer Institute Common Toxicity Criteria, version 3.0 (NCI-CTC 3.0): myelosuppression, palmoplantar erythrodysesthesia (PPED), cardiac, hepatic or renal toxicity grade 3 or 4; and every severe adverse event (SAE) as long as a correlation to the study medication was at least "possible" using standard SAE grading criteria. Each dose level was evaluated for DLTs using the criteria detailed above before advancing to the next level. In both parts of the study, the dosing of TMZ and PEG-Dox was modified as follows: If myelotoxicity of grade 3 or 4 occurred, the next cycle of chemotherapy was delayed, and the dose was reduced to 75% in the next cycle. If grade 4 myelotoxicity recurred after dose reduction or persisted for more than four weeks, treatment was terminated. All patients received trimethoprim-sulfamethoxazole as prophylaxis against *Pneumocystis jiroveci *pneumonia during concomitant treatment with radiotherapy plus TMZ.

### Surveillance and follow-up

The baseline examination included magnetic resonance imaging (MRI, minimum T1, T1 plus gadolinium and FLAIR), full blood counts and blood chemistry tests, and a physical examination. During radiotherapy, patients were seen weekly. Transthoracic echocardiography was performed before the first application of PEG-Dox and at every 4^th ^cycle of the maintenance chemotherapy. During and after radiotherapy, full blood counts were drawn every week until termination of the regimen. 28 days after the completion of radiotherapy and every 8 weeks thereafter, clinical data and KPS were raised prior to every cycle throughout the study, until the tumor progressed. Patients in whom toxicity precluded further treatment were closely followed up until resolution or stabilization of the respective condition. Standard supportive care measures were thoroughly applied in addition to chemotherapy. At tumor progression, patients were treated at the investigator's discretion. The therapeutic modalities used in recurrent disease included re-resections, re-radiotherapy, and salvage chemotherapy (e.g. CCNU, and Imatinib Mesylate based chemotherapies).

### Evaluation of Toxicity and Activity

Tumor progression was defined according to Macdonald criteria [15]. Toxicity (dose limiting toxicity, DLT, and targeted dose) graded according to NCI-CTC 3.0 in phase I and progression free survival at 12 months (PFS-12) after initiation of therapy in phase I and II were defined as the primary endpoints of the study; PFS-24, median overall survival (mOS), overall survival after 1 and 2 years (OS-12, OS-24), median time to progression (mTTP), response rate (RR, complete responses plus partial responses), rate of stabilizations (SD, stable disease), and the toxicity profile were secondary endpoints.

### MGMT Evaluation

The *MGMT *methylation status was determined by the classic gel based methylation specific PCR (MSP) assay [4] and a high throughput quantitative MSP technique (qMSP) as part of a validation study [16].

### Statistical Methods

The study was planned on an adapted intent-to-treat-design. An interim analysis was performed after phase I (termination criterion: unacceptable toxicity) of the study and after treating 10 patients in phase II (termination criterion: tumor progression within 10 weeks in more than 8 patients). The primary endpoint of the study was progression free survival probability at 12 months starting at the time of diagnosis. The trial was designed to accrue 60 patients (plus 5% drop-out reserve) with glioblastoma and to detect an improvement of the PFS-12 of 15.6% as compared to the data of the EORTC26981/NCIC-CE.3 (radiotherapy plus concomitant and adjuvant TMZ) arm (PFS-12: 26.9%) with an α error of 0.10 and β error of 10%. The exact patient number was determined during study accrual after publication of the EORTC26981/NCIC-CE.3 trial in 2005. Based on a Fleming design, the hypothesis tested was H_0_:PFS-12 ≤ 26.9% and H_1_: PFS-12 ≥ 42.5% The minimum PFS-12 to consider the study as "positive" was 36.7%.

As this trial was not randomized, the EORTC26981/NCIC-CE.3 data were used as historical controls and the Cox proportional hazard model was fit to directly assess the effect of the new treatment measured by their Hazard Ratios (HR) in presence of known prognostic factors (age, KPS, extent of surgical resection, corticosteroids at beginning of therapy) in order to correct for selection biases. HR, median PFS and OS, PFS-12, OS-12, and OS-24 were assessed in all patients and in subsets split by MGMT methylation status. The Cox proportional hazard model was fit to assess the prognostic value of the methylation status of the MGMT promoter in combination with the protocol treatment modalities.

## Results

### Patient Characteristics

In total, 70 patients were screened and 63 patients (40 male, 23 female) with newly diagnosed glioblastoma were included and evaluable according to the intent-to-treat design (Figure 1). Median age was 54 years (range: 30-73) and 40% were older than 60 years. The median KPS at inclusion was 90% (range: 70-100%) for the total population; 29% of the patients were on steroids at entry into the study (Table 1).

**Table 1:** Baseline characteristics of the RNOP-09 cohort as compared to the patients enrolled into the EORTC 26981/NCIC-CE.3 trial.

Baseline characteristics		
	**Treatment**	
	
	**RNOP-09** **(N = 63)**	**EORTC/NCI-C/NCI-C** **(N = 287)**

	**N (%)**	**N (%)**

**Sex**		
Female	23 (36.5)	102 (35.5)
Male	40 (63.5)	185 (64.5)

**Age (class)**		
<= 50	19 (30.2)	95 (33.1)
51-60	19 (30.2)	109 (38.0)
>60	25 (39.7)	83 (28.9)

**Performance status (KPS)**		
90-100%	22 (34.9)	113 (39.4)
80%	33 (52.4)	136 (47.4)
70%	8 (12.7)	38 (13.2)

**Extent of surgery**		
Complete	28 (44.4)	113 (39.4)
Partial	23 (36.5)	126 (43.9)
Biopsy	9 (14.3)	48 (16.7)
Not recorded	3 (4.8)	0 (0.0)

**Corticosteroids at study entry**		
No	39 (61.9)	94 (32.8)
Yes	18 (28.6)	193 (67.2)
Missing data	6 (9.5)	0 (0.0)

**MGMT promoter**		
Methylated	16 (25.4)	46 (16.0)
Unmethylated	17 (27.0)	60 (20.9)
Missing	30 (47.6)	181 (63.1)

**Survival at evaluation**		
Alive	22 (34.9)	33 (11.5)
Dead	41 (65.1)	254 (88.5)

**Progression free survival at evaluation**		
Not progressive and alive	7 (11.1)	15 (5.2)
Progressive or dead	56 (88.9)	272 (94.8)

51 patients (81%) had undergone open surgery at primary diagnosis, aiming at maximum tumor removal. All patients received conventional involved-field radiotherapy with a total dose of 60 Gy and concomitant TMZ (75 mg/m2 daily, every day including weekends) during radiotherapy. Three of the patients did not complete the combined radio-chemotherapy due to progressive disease or were lost for follow-up but were included in the analysis due to the intent-to-treat design.

The most important prognostic risk factors (age, KPS, extent of resection, corticosteroids at entry) did only moderately differ between this study and the EORTC26981/NCIC-CE.3 trial (Table 1). Based on sufficient patient numbers, we concluded that a comparison of our data to the EORTC26981/NCIC-CE.3 trial was feasible.

### Toxicity of the combination regimen

During phase I, the escalation groups consisted of 7 (5 mg/m^2 ^PEG-Dox), 4 (10 mg/m^2^), 3 (15 mg/m^2^) and 4 (20 mg/m^2^) patients. One grade 4 leukopenia and neutropenia occurred in the first group within 3 months after diagnosis which was therefore expanded from 4 to 7 patients in accordance to the treatment plan. In the 2^nd^, 3^rd ^and 4^th ^treatment groups, the regimen was tolerated without DLT. As no DLT was observed in dose group 4, the targeted dose was reached and we proceeded to the efficacy phase of the trial with PEG-Dox in a dose of 20 mg/m^2^.

In the summarized toxicity data from phase I and II, the most frequent adverse event attributable to PEG-Dox was palmaroplantar erythrodysesthesia (PPED) occurring in almost all patients to at least some degree. Still, severe cutaneous side effects were rare. During the administration of 402 PEG-Dox infusions, grade 3 or 4 toxicities - defined as bullous exanthema - were seen in only 4 patients. Occurrence of PPED was reduced by oral pyridoxine in a daily dose of 3 × 100 mg, low-dose oral corticosteroids, and cool pads during the infusion of PEG-Dox [17]. The most common side effect attributable to a combined effect of TMZ and PEG-Dox was myelosupression (Table 2). Grade 3 to 4 leukopenia was observed in 12 patients (19%, EORTC26981/NCIC-CE.3: 7%), grade 3 and 4 thrombocytopenia occurred in 7 patients (11%, EORTC26981/NCIC-CE.3: 12%). Grade 3 and 4 lymphopenia, the most common haematologic toxicity, occurred in 33 patients (52%, EORTC26981/NCIC-CE.3: no data available). Despite this high proportion of lymphopenia, only two patients suffered from opportunistic *Pneumocystis jierovici *pneumonia (one NCI-CTC 3.0 grade 4 event), while 7 patients (15.2%) were treated with community-acquired pneumonia that was only possibly related to the ongoing chemotherapy. As in the other published studies with PEG-Dox in high-grade gliomas [10-13], we did not observe cardiotoxic side effects, even in cases with cumulative doses of up to 550 mg/m^2^. Two patients developed deep vein thrombosis. Overall, 58 SAEs occurred, and 9 of these SAEs were at least possibly related to PEG-Dox. Two patients died due to a possibly treatment-related complication (one pulmonary embolism, one unclear rapid decline in general condition). The toxicity data are summarized in Table 2.

**Table 2:** Side effects of the combined radio-chemotherapy with TMZ and PEG-Dox.

Treatment related toxicity **			
	**Common toxicity criteria (NCI CTC Version 3.0)**		
	
	**RNOP-09** **Grade 3**	**RNOP-09** Grade 4*	**EORTC/NCI-C** **Grade 3+4**

	**N (%)**	**N (%)**	**N (%)**

**Gastrointestinal**			
Vomitus/nausea	4 (6.3)	0 (0.0)	6 (2)
Stomatitis	2 (3.2)	0 (0.0)	not reported
Gastritis	2 (3.2)	0 (0.0)	not reported
Diarrhea	3 (4.8)	0 (0.0)	not reported

**Skin**			
PPED/rash	4 (6.3)	0 (0.0)	9 (3)
Herpes simplex infection	2 (3.2)	0 (0.0)	not reported
Edema	2 (3.2)	0 (0.0)	not reported
Anaphylaxis	0 (0.0)	1 (1.5)	not reported

**Infection**			
Pneumonia	8 (12.7)	1 (1.5)	not reported
Wound infection	1 (1.5)	0 (0.0)	not reported
not specified	9 (14.2)	0 (0.0)	20 (7)

**Blood/bone marrow**			
Leukopenia	9 (14.3)	3 (4.7)	20 (7.0)
Lymphopenia	6 (9.5)	27 (43.9)	not reported
Thrombopenia	4 (6.3)	3 (4.7)	33 (12.0)
Anemia	0 (0.0)	2 (3.2)	4 (1.0)

**Cardiac and vascular toxicity**			
Cardiac toxicity	0 (0.0)	0 (0.0)	not reported
Deep vein thrombosis	2 (3.2)	0 (0.0)	not reported
Pulmonary embolism	0 (0.0)	1 (1.5)	not reported

* Two additional patients died, one due to pulmonary embolism and une due to unclear decline in general condition (CTC grade 5).

** Side effects are listed irrespective if they were related to the therapy.

### Activity of the combination regimen

**Figure 2:**
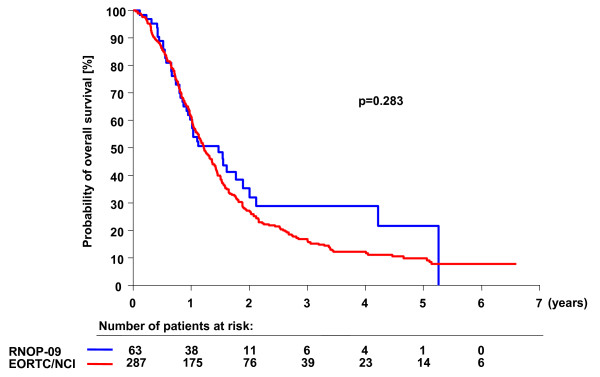
**Overall survival of RNOP-09 patients as compared to historical control**. Kaplan-Meier estimates of overall survival according to treatment group. The unadjusted hazard ratio for death among patients treated with PEG-Dox and prolonged administration of TMZ as compared with those treated in the EORTC26981/NCIC-CE.3 trial was 0.83 (CI: 0.60-1.16; p = 0.28).

All patients were observed until progression after treatment initiation. One patient was lost to follow-up, and his data were included in the statistical analysis using the last date of observation. PFS-12 as primary endpoint was 30.2% as compared to 26.9% for patients treated with combined radio-chemotherapy in the EORTC26981/NCIC-CE.3 study (adjusted HR for progression free survival: 0.91; CI: 0.67-1.26; p = 0.58). The OS-24 was 35.3% (EORTC26981/NCIC-CE.3: 27.2%), the mOS was 17.6 months (EORTC26981/NCIC-CE.3: 14.6 months), and the adjusted HR for overall survival was 0.79 (CI: 0.55-1.14; p = 0.21, Figure 2). Complete response during the protocol therapy including radiotherapy and chemotherapy (CR, n = 2), partial response (PR, n = 3) or stable disease (SD, n = 41) were observed in 73% of patients 8 weeks after initial diagnosis. 8 weeks after initiation of the maintenance therapy (i.e. 20 weeks after tumor resection), 34 patients (53%) were free of progression [18]. There was no significant difference between patients that received less than 20 mg/m^2 ^PEG-Dox in the phase I part of the study and the patients treated with 20 mg/m^2 ^[see Additional File 1]. All other endpoints are summarized in Table 3. Of note, the combination of PEG-Dox, prolonged TMZ, and radiotherapy was significantly superior to radiotherapy alone with respect to overall survival and PFS-12 (30.2% vs. 9.1%; p < 0.002) confirming previous results [2] (data not shown).

**Table 3:** Primary and secondary endpoints of the RNOP-09 study as compared to the EORTC26981/NCIC-CE.3 trial.

	EORTC/NCI-C (N = 287)	RNOP-09 (N = 63)
**All patients**		

*Progression Free Survival*		
Median duration (mo)	6.9 (5.8-8.2)	6.5 (6.0-8.3)
Rate at 1 yr (%) †	26.9 (21.9-32.2)	30.2 (19.4-41.6)
Rate at 2 yrs (%)	11.2 (7.9-15.1)	11.0 (4.4-20.9)
Hazard Ratio ‡	1.00	0.91 (0.67-1.26)
p-value **	P = 0.58	

*Overall Survival*		
Median duration (mo)	14.6 (13.2-16.8)	17.6 (12.2-22.7)
Rate at 1 yr (%)	61.2 (55.3-66.6)	60.3 (47.2-71.2)
Rate at 2 yrs (%)	27.2 (22.2-32.5)	35.3 (22.1-48.7)
Hazard Ratio ‡	1.00	0.79 (0.55-1.14)
p-value **	P = 0.21	

**MGMT methylation status**	(N = 106) (46 meth., 60 unmeth.)	(N = 33) (16 meth., 17 unmeth.)

**Methylated MGMT Promoter**		

*Progression Free Survival*		
Rate at 1 yr (%)	40.0 (25.8-53.8)	31.3 (11.4-53.7)
Hazard Ratio ‡	1.00	0.79 (0.39-1.61)
p-value **	P = 0.52	

*Overall Survival*		
Rate at 1 yr (%)	77.8 (62.6-87.4)	68.8 (40.5-85.6)
Rate at 2 yrs (%)	48.9 (33.7-62.4)	37.5 (15.4-59.8)
Hazard Ratio ‡	1.00	0.98 (0.46-2.11)
p-value **	P = 0.97	

**Unmethylated MGMT Promoter**		

*Progression Free Survival*		
Rate at 1 yr (%)	13.3 (6.2-23.2)	25.5 (7.3-44.9)
Hazard Ratio ‡	1.00	1.03 (0.47-2.26)
p-value **	P = 0.94	

*Overall Survival*		
Rate at 1 yr (%)	56.7 (43.2-68.1)	41.2 (18.6-62.6)
Rate at 2 yrs (%)	14.8 (7.2-25.0)	16.5 (2.9-39.9)
Hazard Ratio ‡	1.00	0.93 (0.44-1.95)
p-value **	P = 0.84	

* Numbers in parenthesis are 95 percent confidence intervals

† Primary endpoint

‡ Obtained from the Cox model after adjustment of RNOP-09 treatment effect for extent of surgery (B/PR/CR), Age (< = 50,51-60,>60), Performance Status (0,1,2), administration of corticosteroids (No/Yes)

** Wald's test

### MGMT methylation status and correlation with tumor progression and survival

The *MGMT *methylation status could be determined in 33 patients (52%). The samples with assignable MGMT promoter status were representative of the overall treatment population with respect to prognostic factors and outcomes except for a slight imbalance in the resection status (patients with determined MGMT promoter status had less biopsies, data not shown). Of the evaluated tumors, 16 (48%) had detectable MGMT promoter methylation, whereas 17 (52%) did not. The proportion of MGMT methylated and unmethylated tumors is similar to previous studies [4]. With respect to all analyzed endpoints, there were no statistically significant differences between both groups. However, the statistical power of this study was too low to allow final conclusions (Table 3).

## Discussion

This is the first phase II-trial to evaluate the activity of PEG-Dox and prolonged administration of TMZ in addition to standard radio-chemotherapy in the first-line treatment of patients with glioblastoma. Our data were compared to the standard-of-care established in the EORTC26981/NCIC-CE.3 trial by means of a historical control analysis to disclose superiority of our combined approach. In this multi-institutional trial, we could show that neither the addition of PEG-Dox (20 mg/m^2 ^day 1 and 15 of 28 days) nor the prolonged administration of TMZ to standard therapy comprising TMZ and radiotherapy meaningfully improves PFS-12 in comparison to the EORTC study. However, the study was not formally powered to evaluate long-term survival. Although the combined therapy slightly increased the median overall survival of the total study population to 17.6 months as compared to 14.6 in the EORTC26981/NCIC-CE.3 study [2], this difference did not reach statistical significance. Agreed confidence intervals of medians were largely overlapping (RNOP-09: median 14.6; CI: [13.2-16.8] - EORTC26981/NCIC-CE.3: median: 17.6; CI: [12.2-22.7]). In addition, 73% of all patients had objective responses or stabilizations according to Macdonald's criteria [15].

The overall toxicity pattern was acceptable over the study course. As expected, the most common adverse effect clearly attributable to PEG-Dox was palmaroplantar erythrodysesthesia (PPED). The relatively high incidence of PPED under therapy with PEG-Dox may be explained by accumulation of liposomes in the small capillaries of the skin [19,20]. Higher grade toxicity was rare due to extensive supportive care measures [17] and in line with previous studies suggesting that skin toxicity occurs more frequently if the dose exceeds 60 mg/m^2 ^per month [20,21]. Hematological side effects were pronounced as compared to the EORTC26981/NCIC-CE.3 study and possibly caused more infectious complications. Although the data cannot be compared directly as they were evaluated in different study populations, they suggest that the addition of PEG-Dox and/or prolonged administration of TMZ increased the myelotoxicity of TMZ moderately. Still, the overall toxicity profile remained favorable. In line with previous reports, cardiotoxicity did not occur [22].

Although unknown biasing factors cannot be excluded due to the lack of randomization, our data allow final conclusions because of the large patient population (63 patients), the statistical design (power of 90% to detect a meaningful improvement of PFS-12), and the well balanced patient populations of RNOP-09 and the EORTC26981/NCIC-CE.3 study. The slight increase of long term survivors is low as compared to other phase II studies [23] but may also reflect minor differences in the patients characteristics (e.g. low proportion of patients on steroids at entry). Thus, it is unlikely that a larger patient population, e.g. in a Phase III-setting, would unveil a clinically meaningful improvement of the PFS-12 or the proportion of long term survivors by PEG-Dox. In addition, our data does not support prolonged TMZ based chemotherapy schedule in non-progressive patients. Although non-progressive patients were treated in the RNOP-09 trial up to at least 12 cycles of maintenance chemotherapy with TMZ, this neither translated into a meaningful improvement of PFS nor mOS, at least summarized over all patients. The slightly increased toxicity of the combined therapy did not translate into a relevant PEG-Dox related mortality. Thus it appears unlikely that PEG-Dox induced toxicity masked the positive effects of prolonged TMZ administration. With the comparable activity, the common cutaneous side effects, and the increased rate of infections this combined therapy did not show sufficient signs of activity in addition to the EORTC26981/NCIC-CE.3 protocol to be recommended.

## Conclusion

Based on *in vitro *results, PEG-Dox was among the promising substances of a large panel of "classical" chemotherapeutic substances tested in glioblastoma during the last decades [24]. None of these substances, except TMZ, could improve the prognosis of glioblastoma in a first-line setting, and the improvement is largely limited to patients with a methylated MGMT promoter. Recent trials that tested dose intensifications of TMZ [25,26] or combinations of different agents with TMZ [23,27] failed to improve the prognosis of patients with an unmethylated MGMT promoter. Thus, we suggest that combinations of classic cytostatic drugs may not be effective to improve the outcome of these patients. New targeted therapies combined with cytotoxic drugs might be better suitable to delay progression and improve survival in patients with an unmethylated MGMT promoter. This hypothesis will have to be tested in randomized phase II trials, as even large non-randomized trials might miss clinically meaningful results due to the inappropriate control for unknown biasing factors beyond the known prognostic factors. Such studies are in development or started accrual recently and hopefully will produce relevant improvements for these patients.

## Competing interests

Christoph P. Beier: travel support by Schering-Plough; unrestricted educational grant by Schering-Plough.

Ulrich Bogdahn: travel support by Schering-Plough; scientific advisor and speaker for Schering-Plough.

Peter Hau: travel support by Schering-Plough; scientific advisor and speaker for Schering-Plough; several unrestricted educational grants by Schering-Plough.

## Authors' contributions

CPB: writing of the manuscript, evaluation of data, statistical analysis, accrual of patients. CS: compilation and evaluation of patient data. TG: statistical evaluation, writing of the manuscript. CK: accrual of patients, compilation of patient data. DB: accrual of patients, writing of the manuscript. OG: protocol writing, accrual of patients, organization of translational data. AS: protocol writing, accrual of patients, writing of the manuscript. BH: protocol writing, accrual of patients, compilation of patient data. AB: accrual of patients, provision of patient material, compilation of follow-up data. CD: statistical planning. TJ: protocol writing, accrual of patients, evaluation of patient data. OK: accrual of patients, compilation of follow-up data. TP: protocol writing, neuropathological review of the samples, writing of the manuscript. MP: accrual of patients, provision of patient material, compilation of follow-up data. PR: protocol writing, neuropathological evaluation of the tumor samples. AM: accrual of patients, compilation of follow-up data. GS: accrual of patients, compilation of follow-up data, writing of the manuscript. MH: MGMT promoter analysis, writing of the manuscript. UB: protocol writing, design of the trial, accrual of patients. PH: protocol writing, design of the trial, accrual of patients, data evaluation, writing of the manuscript, final manuscript approval, vouches for the correctness of the published data

## Pre-publication history

The pre-publication history for this paper can be accessed here:

http://www.biomedcentral.com/1471-2407/9/308/prepub

## Supplementary Material

Additional file 1 **Comparison of patients treated in phase I or II**. Relative progression free survival of patients treated with less than 20 mg/m^2 ^during the phase I part of RNOP-09 as compared to patients treated with 20 mg/m^2 ^in the phase I and phase II part (p = 0.218, Log-rank-test). Click here for file
