# Supplementary figures and images for: RNOP-09: Pegylated liposomal doxorubicine and prolonged temozolomide in addition to radiotherapy in newly diagnosed glioblastoma - a phase II study
Source: BMC Cancer. 2009 Sep 2;9:308. doi: 10.1186/1471-2407-9-308 (PMC2749868; doi:10.1186/1471-2407-9-308)

## Slide 1
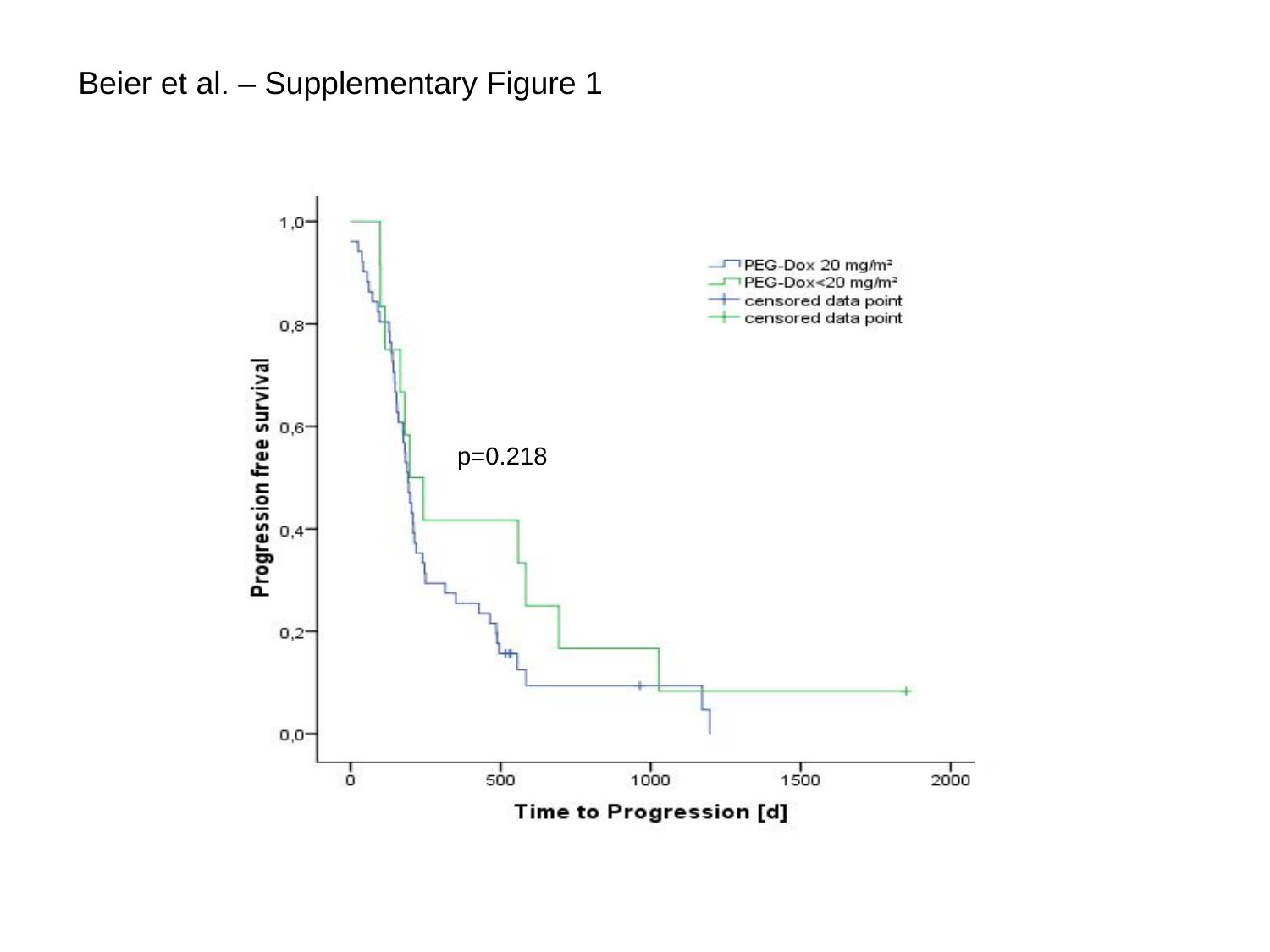

Beier et al. – Supplementary Figure 1
p=0.218

Supplement: Additional file 1 — Comparison of patients treated in phase I or II. Relative progression free survival of patients treated with less than 20 mg/m2 during the phase I part of RNOP-09 as compared to patients treated with 20 mg/m2 in the phase I and phase II part (p = 0.218, Log-rank-test). [file 1471-2407-9-308-S1.ppt]
